# Supplementary material for: Afatinib and Dacomitinib Efficacy, Safety, Progression Patterns, and Resistance Mechanisms in Patients with Non-Small Cell Lung Cancer Carrying Uncommon EGFR Mutations: A Comparative Cohort Study in China (AFANDA Study)
Source: Cancers (Basel). 2022 Oct 28;14(21):5307. doi: 10.3390/cancers14215307 (PMC9656097; doi:10.3390/cancers14215307)
Supplement: Supplementary file 1 [file cancers-14-05307-s001.zip › cancers-1969058-supplementary.pdf]

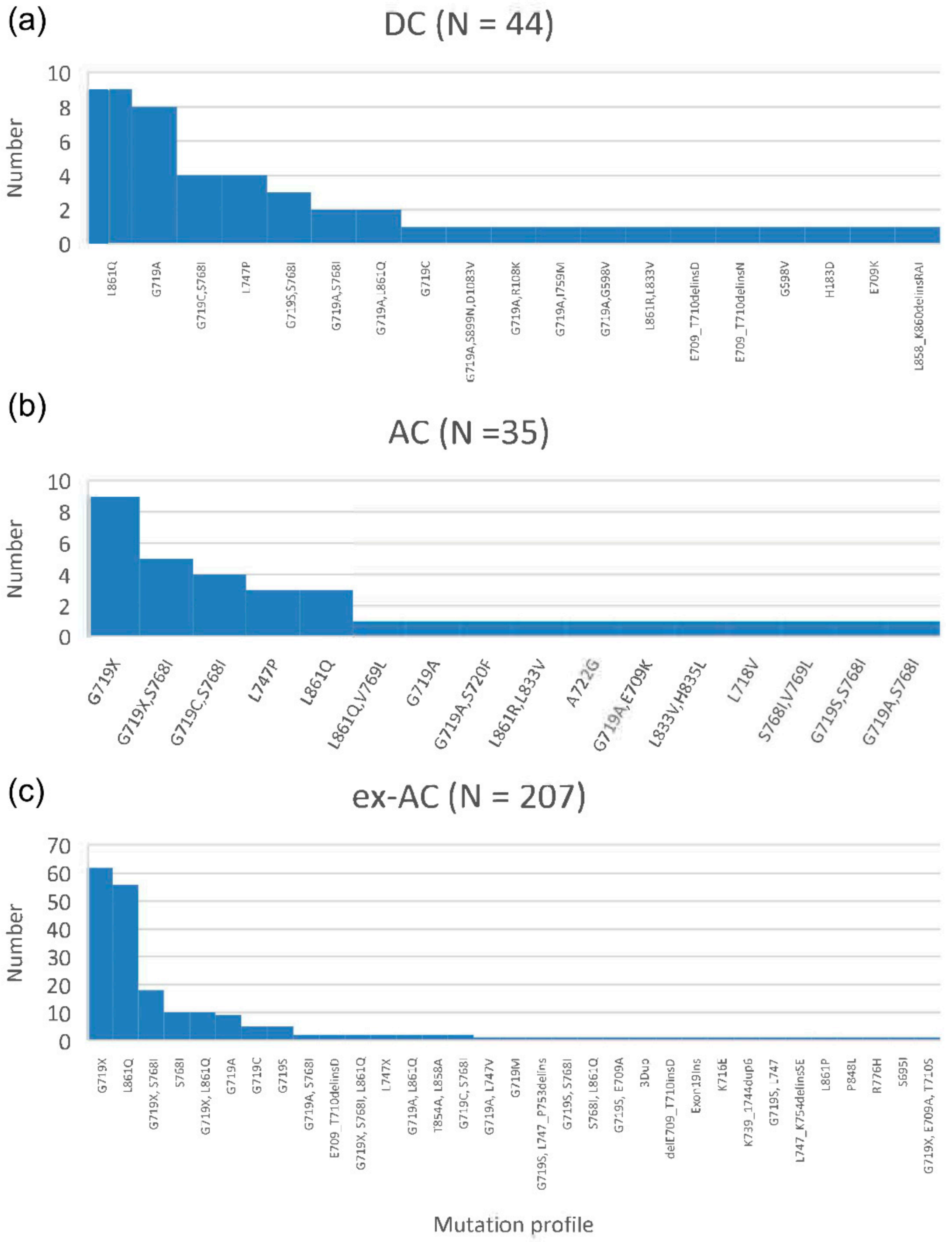

Figure S1. The mutation landscape of the three cohorts (DC, AC, and ex-AC) in this study. (**a**) DC, dacomitinib cohort; (**b**) AC, afatinib cohort; (**c**) ex-AC, external afatinib cohort.

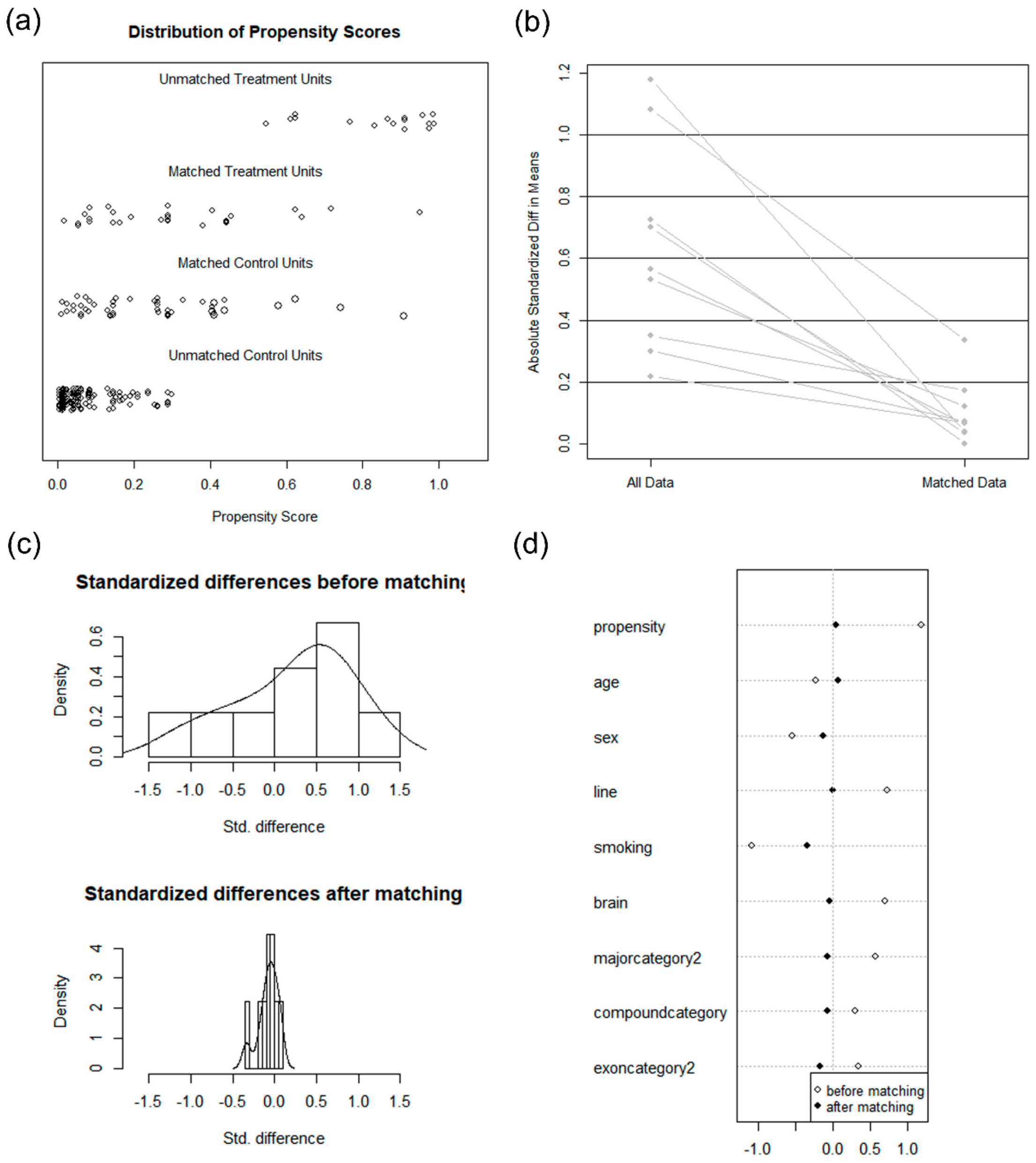

Figure S2. Balance analyses between DC and ex-AC after PSM. (**a**) DC, dacomitinib cohort; (**b**) ex-AC, external afatinib cohort; (**c**) TKI, tyrosine kinase inhibitor; (**d**) PSM, propensity score matching.

Table S1. Univariate and multivariate analyses of PFS in the combined cohort of DC and ex-AC after PSM (N = 76)

Characteristics	N	Univariate analysis		Multivariate analysis		
		Median (months)	*P* value	HR	95% CI	*P* value
**Intervention**			**0.033**			**0.010**
Dacomitinib/Afatinib	28/48	15.9/10.4		3.030	1.304-7.042	
**Age**			0.197			0.111
＜60/≥60	31/45	16.6/12.0		1.758	0.878-3.521	
**Sex**			0.297			0.420
Female/Male	48/28	11.5/15.9		0.739	0.353-1.544	
**Treatment history**			0.713			0.449
TKI-naïve/pretreated	68/8	15.4/12.0		1.480	0.536-4.087	
**Smoking history**			0.358			**0.047**
No	45	15.9		Reference	-	-
Yes	25	10.5		2.864	1.238-6.626	0.014
Unknown	6	16.6		1.361	0.416-4.454	0.611
**Brain metastases**			0.762			0.363
No/Yes	60/16	14.2/10.9		0.667	0.279-1.595	
**Mutation category**			0.307			0.097
G719X	14	10.4		Reference	-	-
L861X	19	8		1.234	0.484-3.147	0.660
S768I	3	20.7		0.222	0.043-1.151	0.073
Others	15	12.5		0.736	0.257-2.108	0.568
Compound mutations	25	16.6		0.504	0.192-1.319	0.163

Data were shown as n (%). PFS, progression-free survival; DC, dacomitinib cohort; ex-AC, external afatinib cohort; TKI, tyrosine kinase inhibitor; PSM, propensity score matching. Set variables before "/" as reference.

Table S2. Progression patterns of DC and AC

Progression patterns	DC (N = 20)	AC (N = 25)	*P* value
Intracranial failure	4 (21.1)	12 (50)	**0.002**
Intrathoracic failure	10 (52.6)	2 (8.3)	
Intracranial+intrathoracic failure	1 (5.3)	6 (25)	
Intracranial+extrathoracic failure	0 (0)	3 (12.5)	
Extrathoracic*	5 (26.3)	2 (8.3)	

*Extrathoracic, not including intracranial failure. DC, dacomitinib cohort; AC, afatinib cohort.

Table S3. Secondary T790M mutation detected after progression in DC and AC

Characteristics	DC (N = 13)	AC (N = 17)	*P* value
T790M (+)	2 (15.4)	2 (11.8)	0.772
T790M (-)	11 (84.6)	15 (88.2)	

2G TKIs, second-generation tyrosine kinase inhibitors; DC, dacomitinib cohort; AC, afatinib cohort.

Table S4. Briging local interventions after progression in DC and AC

Local interventions*	DC (N = 20)	AC (N = 25)	*P* value
Yes	2 (10)	14 (56)	**0.001**
No	18 (90)	11 (44)	

*Local interventions, including brain radiotherapy, and local interventional therapies. DC, dacomitinib cohort; AC, afatinib cohort.

Table S5. A summary of published cases with NSCLC harboring uncommon *EGFR* mutations treated with dacomitinib

First author	Type	Region	N	Sex	Age	Histology	LOT	Mutation subtype	Best response	ORR	PFS
Zhang [1]	R	China	1	Female	62	AC	1	G719A	PR	NA	6.6
			1	Female	64	AC	1	L861Q	non-CR/non-PD	NA	10+
Reckamp [2]	P	USA	1	NA	NA	AC	1	G719C+S768I	PR	NA	15.5
Peng [3]	R	China	1	Female	50	AC	1	G719A+I706T	PR	NA	1+
Park [4]	P	Korea	1	NA	NA	AC	2	G719X	PR	NA	NA
Morita [5]	R	Japan	1	Female	71	AC	6	G719A	PR	NA	7.8
Li [6]	R	China	11	NA	NA	AC	2~4	G719X, S768I, L861Q, L747P	NA	54.5%	10.3
Choudhury [7]	P	USA	1	NA	NA	AC	2	G719A	PR	NA	17+
Biswas [8]	CA	India	2	NA	NA	NA	NA	G719X	PR	NA	NA
			1	NA	NA	NA	NA	L861Q	PR	NA	NA
Jänne [9]	P	USA	1	NA	NA	AC	1	E709A+G719S	PR	NA	NA
Li [10]	P+R	China	32	Female (62.5%)	Median: 64	31 AC, 1 ASC	Median (range): 1 (1~6)	G719X, S768I, L861X	NA	56.3%	10.3
			18	NA	NA	NA	1	G719X, S768I, L861X	NA	72.2%	UR
			14	NA	NA	NA	Later-line	G719X, S768I, L861X	NA	NA	NA

NSCLC, non-small cell lung cancer; *EGFR*, epidermal growth factor receptor; R, retrospective study; P, prospective study; CA, conference abstract; N, number; AC, adenocarcinoma; ASC, adenosqumaous carcinoma; NA, not applicable/not available; EX20ins, *EGFR* 20 exon insertion mutation; LOT, line of therapy; ORR, objective response rate; CR, complete response; PR, partial response; SD, stable disease; PD, progressive disease; PFS, progression-free survival; UR, unreached.

Refefences

1. Zhang J, Wang Y, Liu Z, Wang L, Yao Y, Liu Y, et al. Efficacy of dacomitinib in patients with EGFR-mutated NSCLC and brain metastases. *Thorac Cancer.* 2021;12(24):3407-3415.
2. Reckamp KL, Giaccone G, Camidge DR, Gadgeel SM, Khuri FR, Engelman JA, et al. A phase 2 trial of dacomitinib (PF-00299804), an oral, irreversible pan-HER (human epidermal growth factor receptor) inhibitor, in patients with advanced non-small cell lung cancer after failure of prior chemotherapy and erlotinib. *Cancer.* 2014;120(8):1145-1154.
3. Peng W, Pu X, Jiang M, Wang J, Li J, Li K, et al. Dacomitinib induces objective responses in metastatic brain lesions of patients with EGFR-mutant non-small-cell lung cancer: A brief report. *Lung cancer (Amsterdam, Netherlands).* 2021;152:66-70.
4. Park K, Cho BC, Kim DW, Ahn MJ, Lee SY, Gernhardt D, et al. Safety and efficacy of dacomitinib in korean patients with KRAS wild-type advanced non-small-cell lung cancer refractory to chemotherapy and erlotinib or gefitinib: a phase I/II trial. *J Thorac Oncol.* 2014;9(10):1523-1531.
5. Morita A, Hosokawa S, Yamada K, Umeno T, Kano H, Kayatani H, et al. Dacomitinib as a retreatment for advanced non-small cell lung cancer patient with an uncommon EGFR mutation. *Thorac Cancer.* 2021;12(8):1248-1251.
6. Li HS, Zhang JY, Yan X, Xu HY, Hao XZ, Xing PY, et al. A real-world study of dacomitinib in later-line settings for advanced non-small cell lung cancer patients harboring EGFR mutations. *Cancer Med.* 2022;11(4):1026-1036.
7. Choudhury NJ, Makhnin A, Tobi YY, Daly RM, Preeshagul IR, Iqbal AN, et al. Pilot Study of Dacomitinib for Patients With Metastatic EGFR-Mutant Lung Cancers With Disease Progression After Initial Treatment With Osimertinib. *JCO Precis Oncol.* 2021;5.
8. Biswas B, Ganguly S, Ghosh J, Roy S, Bakshi R, Dabkara D. Real-world experience of dacomitinib in EGFR mutated advanced NSCLC: A single center experience from India. *Journal of Clinical Oncology.* 2021;39(15 SUPPL).
9. Jänne PA, Ou SI, Kim DW, Oxnard GR, Martins R, Kris MG, et al. Dacomitinib as first-line treatment in patients with clinically or molecularly selected advanced non-small-cell lung cancer: a multicentre, open-label, phase 2 trial. *The Lancet Oncology.* 2014;15(13):1433-1441.
10. Li H-S, Yang G-J, Cai Y, Li J-L, Xu H-Y, Zhang T, et al. Dacomitinib for Advanced Non-small Cell Lung Cancer Patients Harboring Major Uncommon EGFR Alterations: A Dual-Center, Single-Arm, Ambispective Cohort Study in China. *Frontiers in Pharmacology.* 2022;13.
